# Characteristics, comorbidities and survival analysis of young adults hospitalized with COVID-19 in New York City

**DOI:** 10.1371/journal.pone.0243343

**Published:** 2020-12-14

**Authors:** Brian L. Altonen, Tatiana M. Arreglado, Ofelia Leroux, Max Murray-Ramcharan, Ryan Engdahl

**Affiliations:** 1 Division of Population Health and Research Administration, NYC Health + Hospitals, New York, New York, United States of America; 2 Division of Clinical Informatics, NYC Health + Hospitals, Harlem Hospital, New York, New York, United States of America; 3 Department of Surgery, NYC Health + Hospitals, Harlem Hospital, New York, New York, United States of America; 4 Department of Surgery, NYC Health + Hospitals, Harlem and Woodhull Hospitals, New York, New York, United States of America; University of South Carolina, UNITED STATES

## Abstract

This study reviewed 395 young adults, 18–35 year-old, admitted for COVID-19 to one of the eleven hospitals in New York City public health system. Demographics, comorbidities, clinical course, outcomes and characteristics linked to hospitalization were analyzed including temporal survival analysis. Fifty-seven percent of patients had a least one major comorbidity. Mortality without comorbidity was in 3.8% patients. Further investigation of admission features and medical history was conducted. Comorbidities associated with mortality were diabetes (n = 54 deceased/73 diagnosed,74% tested POS;98.2% with diabetic history deceased; Wilcoxon p (*Wp*) = .044), hypertension (14/44,32% POS, 25.5%; *Wp* = 0.030), renal (6/16, 37.5% POS,11%; *Wp* = 0.000), and cardiac (6/21, 28.6% POS,11%; *Wp* = 0.015). Kaplan survival plots were statistically significant for these four indicators. Data suggested glucose >215 or hemoglobin A1c >9.5 for young adults on admission was associated with increased mortality. Clinically documented respiratory distress on admission was statistically significant outcome related to mortality (*X*^2^ = 236.6842, df = 1, p < .0001). Overall, 28.9% required supportive oxygen beyond nasal cannula. Nasal cannula oxygen alone was required for 71.1%, who all lived. Non-invasive ventilation was required for 7.8%, and invasive mechanical ventilation 21.0% (in which 7.3% lived, 13.7% died). Temporal survival analysis demonstrated statistically significant response for Time to Death <10 days (*X*^2^ = 18.508, df = 1, p = .000); risk lessened considerably for 21 day cut off (*X*^2^ = 3.464, df = 1, p = .063), followed by 31 or more days of hospitalization (*X*^2^ = 2.212, df = 1, p = .137).

## Introduction

Since the first report from China in December 2019 of SARS-CoV-2, the virus that causes COVID-19 [1], studies have demonstrated clinical characteristics and outcomes of patients in outbreaks in many urban areas [2]. Although COVID-19 is more likely to affect older individuals, information on characteristics and risk factors associated with COVID-19 in young adults is limited. We studied young adults, ages 18 to 35 year-old, who were admitted with COVID-19 in New York City public health system and describe demographics, comorbidities, clinical outcomes, and characterize this population using a survival analysis.

## Materials and methods

### Study setting

A retrospective study was conducted on confirmed COVID-19 in young adults admitted to New York City public hospitals. New York City public hospitals, NYC Heath + Hospitals, encompasses eleven hospitals in four different boroughs of New York City with two in Queens (Elmhurst Hospital and Queens Hospital) and three in Manhattan (Bellevue Hospital, Metropolitan Hospital, and Harlem Hospital), in Brooklyn (Kings County Hospital, Coney Island Hospital and Woodhull Hospital) and in the Bronx (Jacobi Medical Center, Lincoln Hospital and North Central Bronx Hospital).

### Data collection

The study was approved by Institutional Review Board the Biomedical Research Alliance of New York (BRANY) and waiver for informed consent and HIPAA was granted. Data was collected from an integrated electronic medical records system (Epic Health Systems, Verona, WI). As the data is dynamic and constantly evolving in the medical records, we decided to finalize all collected required data on April 30th 2020 for March 5th to April 25th 2020, which includes the exponential phase and time following of COVID-19 pandemic in New York. For the patient records used in this retrospective study, all were fully anonymized for the data accession. Manual individual chart reviews were done by opening all of the collected records to confirm all data including confirmation of a COVID-19 admission for known symptoms of the disease of viral illness including fever or chills, shortness of breath, nausea or diarrhea, as well as co-morbidities, laboratories, and patient characteristics including age, sex, race, body mass index, as well as length of stay and outcomes including mortality (Tables 1–4). A positive COVID-19 test was defined by a positive result on a reverse-transcriptase–polymerase-chain-reaction (RT-PCR) assay of a specimen collected on a nasopharyngeal or oropharyngeal swab and with the aforementioned symptoms.

**Table 1:** Characteristics of young adults (mean age 29.2) admitted with COVID-19 (N, %).

	**Patients by age group, Number of Patients (%)**						
	Total	Alive			Deceased		
		18–23	24–29	30–35	18–23	24–29	30–35
Patients	395 (100)	40 (10.1)	93 (23.5)	207 (52.4)	3 (0.8)	19 (4.8)	33 (8.4)
Gender (n = 395)							
Female	130 (32.9)	16 (4.1)	33 (8.4)	68 (17.2)		5 (1.3)	8 (2.0)
Male	264 (66.8)	17 (4.1)	60 (15.2)	138 (34.9)	3(0.8)	14 (3.5)	25 (6.3)
Unidentified	8 (2.3)	7 (2.0)		1 (0.3)			
Pregnant (n = 22) ^							
No comorbidities	13 (59.1)	2 (9.1)	6 (27.3)	5 (22.7)	0 (0)	0 (0)	0 (0)
With comorbidities	9 (40.9)	1 (4.5)	3 (13.6)	4 (18.2)	0 (0)	0 (0)	1 (4.5)
Race or Ethnicity (n = 395)							
American Indian or Alaskan	1 (0.3)			1 (0.3)			
Asian	17 (4.3)		6 (1.5)	11 (2.8)			
Black	78 (19.7)	7 (1.8)	19 (4.8)	40 (10.1)	1 (0.3)	5 (1.3)	6 (1.5)
Declined	6 (1.5)			5 (1.3)			1 (0.3)
Hispanic	50 (12.7)	3 (0.8)	16 (4.1)	23 (5.8)	1 (0.3)	2 (0.5)	5 (1.3)
Other*	228 (57.7)	28 (7.1)	49 (12.4)	119 (30.1)	1 (0.3)	12 (3)	19 (4.8)
White	15 (3.8)	2 (0.5)	3 (0.8)	8 (2)			2 (0.5)
Smoking Status (n = 395)							
Smoker	39 (9.9)	5 (1.3)	7 (1.8)	24 (6.1)		1 (0.3)	2 (0.5)
Former Smoker	15 (3.8)		3 (0.8)	10 (2.5)		1 (0.3)	1 (0.3)
Non Smoker	253 (64.1)	25 (6.3)	56 (14.2)	131 (33.2)	2 (0.5)	16 (4.1)	23 (5.8)
Smoking status not indicated	88 (22.3)	10 (2.5)	27 (6.8)	42 (10.6)	1 (0.3)	1 (0.3)	7 (1.8)
BMI (n = 354; 89.7% of 395)**							
Underweight (< 18)**	3 (0.8)	1 (0.3)		2 (0.6)			
Normal Weight (18.5–24.9)**	49 (13.8)	7 (2.0)	19 (5.4)	16 (4.5)	1 (0.3)	2 (0.6)	4 (1.1)
Overweight (25.0–29.9)**	118 (33.3)	6 (1.7)	32 (9.0)	70 (19.8)		2 (0.6)	8 (2.3)
Class I Obesity (30.0–34.9)**	74 (20.9)	7 (2.0)	14 (4.0)	46 (13.0)	1 (0.3)	4 (1.1)	2 (0.6)
Class II Obesity (35.0–39.9)**	38 (10.7)		9 (2.5)	20 (5.6)	1 (0.3)	2 (0.6)	6 (1.7)
Class III Obesity (≥ 40)**	72 (20.3)	8 (2.3)	13 (3.7)	33 (9.3)		8 (2.3)	10 (2.8)
No BMI given or calculated#	41 (10.3#)	11 (26.8)	6 (14.6)	20 (48.8)	0 (0)	1 (2.4)	3 (7.3)

^denominator = 22, 8.3% of 130 females

*Includes Hispanic and Latino

**these percentages calculated using n = 354

#denominator for ‘no BMI’ = 41 (10.3% of 395)

**Table 2:** Clinical characteristics of admitted young adults COVID-19 –vital signs and laboratory measures (median, IQR).

Vital Signs [Median (IQR)]	Alive	Deceased	All Patients
Temperature (°C)	100.2 (2.925)	100.1 (2.675)	100.2 (2.90)
Heart rate (beats per min)	111 (22)	117 (23.75)	111 (22)
Oxygen saturation (%)	94 (5)	94.5 (12.75)	94 (6)
Labs* **[Median (IQR)]**	Alive	Deceased	All Patients
White blood cell count (×10^3^ cells per μL), n = 387	7.59 (4.5)	8.03 (6.49)	7.67 (4.63)
Neutrophil (%), n = 385	77.6 (13.28)	79 (13.1)	77.95 (13.15)
Lymphocyte (%), n = 385	14.75 (11.2)	13.1 (10.7)	14.6 (11.2)
Creatinine (mg/dL), n = 385	0.84 (0.35)	0.97 (0.85)	0.86 (0.40)
Glucose (mg/dL), n = 386	111 (40)	139.5 (176)	113 (49)
Aspartate aminotransferase (U/L), n = 374	44 (42)	87 (139)	46 (50.5)
Alanine aminotransferase (U/L), n = 374	44 (46)	54 (79)	46 (52.75)
Lactate Dehydrogenase (x/x), n = 291	395 (209)	491 (389)	406 (226)
C-reactive protein (mg/L), n = 269	64.74 (128.6)	73 (155)	66.1 (135.39)
Ferritin (ng/mL), n = 261	748 (972)	776.3 (1093)	751 (976.1)
Procalcitonin (ng/mL) n = 326	0.19 (0.28)	0.42 (1.2)	0.21 (0.38)
D-dimer (μg/mL), n = 245	333.5 (337)	453 (1502)	354 (355)
Hgb a1C, n = 85	6.55 (5.4)	10.5 (8.8)	6.6 (6)
High-sensitivity cardiac troponin T (ng/L), n = 53	0.01 (0.03)	0.035 (0.15)	0.02 (0.06)

#Temperature, heart rate, SPO2 at hospital presentation

*median values, IQR, n = number of patients with laboratory

**Table 3:** Clinical characteristics of admitted young adults COVID-19 –ventilation/respiratory support (N, %).

Respiratory Support, number of patients (%)	Alive	Deceased##	Total
Nasal cannula oxygen alone	281 (71.1)		
Non-invasive ventilation (BiPAP/CPAP)	31 (7.8)		
Invasive mechanical ventilation	29 (7.3)	54 (13.7)	83 (21.0)
Days of invasive mechanical ventilation, Average [IQR]	18 [12]	8 [8]	11.4 [10]
Tracheostomy, Extubation/Reintubation	8 (2.3)	0	8 (2.3)

##all deceased received respiratory support beyond nasal cannula oxygen

**Table 4:** Clinical characteristics of admitted young adults COVID-19 –length of stay.

	Patients by age group, No. (%)						
	Alive			Deceased			Total
Length of stay no. (%)	18–23	24–29	30–35	18–23	24–29	30–35	
0–7 Days	26 (6.6)	62 (15.7)	145 (36.7)	1 (0.3)	9 (2.3)	15 (3.8)	258 (65.3)
8–14 Days	10 (2.5)	26 (6.6)	40 (10.1)	0	8 (2)	10 (2.5)	94 (23.8)
15–21 Days	3 (0.8)	3 (0.8)	12 (3)	1 (0.3)	2 (0.5)	3 (0.8)	24 (6.1)
>21 Days	1 (0.3)	2 (0.5)	10 (2.5)	1 (0.3)	0	5 (1.3)	19 (4.8)

### Data analysis

The cases data for this study were evaluated using IBM SPSS Statistics v26. The patient information included demographics, age, BMI, comorbidities, smoking status, specific laboratories and inflammatory markers, vital signs, hospital course, respiratory support, outcomes and disposition. In addition, characteristics linked to COVID-19 hospitalization were collected including admission with pregnancy, or in extremis with cardiac arrest or DKA, respiratory support including ventilator use, extubation, those who received tracheotomy, length of stay (LOS), and post-hospitalization within a long-term care facility. The raw data were evaluated in original form (Tables 1–6) as well as modified for use in correlations and comparative analyses, both of which allow for more targeted linear and correlational analyses of the results (Tables 7–9). Examples of these modified data include datasets designed to define groups or ranges of results, and binomials (i.e. 1 = yes, 0 = no; 1 = > indicator value, 0 = ≤ value). Some of the patient characteristics and comorbidities datasets were collected in binomial form (i.e. Diabetes history: 1 = yes, 0 = no). Age was entered as whole number years. For vitals and laboratory data, both the actual result and a series of ranges in varying units, or binomials (0 = normal, 1 = not normal) were developed for analysis. Hospital course and outcomes data were mostly collected and entered as binomial data and/or text that was modified into binomial data. Date-requiring data such as length of stay (LOS) and dates of initiation and cessation of the respiratory support used to calculate the numbers of days and percentages of care related days, in order to assess the effects and relationships of these steps on patient care in relation to overall outcome. Data were initially evaluated using standard Chi Squared 2 x 2 analysis (p < .05 = significant) for range related binomial dataset and if applicable, Fisher’s Exact test. For variables with small numbers being tested (n<45, or with one or more values <5), a statistically significant result in both Chi Squared and Fisher’s Exact outcome with p< .05 more strongly supports a result with just a Chi Squared outcome for p < .05 on its own.

**Table 5:** Comorbidities in young adults COVID-19 admissions.

Comorbidities by Age Group, Number of Patients (%, for denominator n = 395)							
	Alive (n = 340)			Deceased (n = 55)			All (n = 395)
No comorbidities		151(38.2)			15 (3.8)		166 (42.1)
One or more comorbidities		189(47.8)			40 (10)		229 (57.9)
Age Distributions, years	**18–23**	**24–29**	**30–35**	**18–23**	**24–29**	**30–35**	**Group Sums **
Total Number of Patients (%)	40 (11.8)	93 (27.4)	207 (60.9)	3 (5.5)	19 (34.5)	33 (60.0)	395
No comorbidities	12 (3.0)	49 (12.4)	90 (22.8)	1 (0.3)	7 (1.8)	7 (1.8)	166
***Patients with***							
Asthma	8 (2.0)	16 (4.1)	31 (7.8)		3 (0.8)	5 (1.3)	63
Obstructive sleep apnea		4 (1)	11 (2.8)		2 (0.5)	2 (0.5)	10
Other chronic pulmonary diagnosis		1 (0.3)	2 (0.5)			1 (0.3)	4
Hypertension	2 (0.5)	4 (1)	24 (6.1)		5 (1.3)	9 (2.3)	44
***Diabetes mellitus***							68
Type I diabetes mellitus	1 (0.3)	5 (1.3)	2 (0.5)	1 (0.3)	7 (1.8)	11 (2.8)	
Type II diabetes mellitus	4 (1)	7 (1.8)	24 (6.1)				
Gestational diabetes mellitus in pregnancy			5 (1.3)				
Prediabetes			3 (0.8)				
***Renal diagnoses***							19
Nephrotic syndrome			1 (0.3)				
Chronic kidney disease			1 (0.3)				
Chronic kidney disease, stage 4			1 (0.3)				
Chronic kidney disease, stage 5			1 (0.3)				
End stage renal disease		2 (0.5)	7 (1.8)		1 (0.3)	2 (0.5)	
Chronic kidney disease, unspecified					1 (0.3)	1 (0.3)	
Neuromuscular dysfunction of bladder					1 (0.3)		
***Cardiac diagnoses***							17
Atherosclerotic heart disease			2 (0.5)				
Acute and subacute infective endocarditis						1 (0.3)	
Right bundle-branch block			1 (0.3)				
Pre-excitation syndrome						1 (0.3)	
Atrial fibrillation						1 (0.3)	
Diastolic (congestive) heart failure			2 (0.5)				
Heart Failure		1 (0.3)				1 (0.3)	
Cardiomegaly						1 (0.3)	
Cardiac murmur	1 (0.3)		1 (0.3)				
Ebstein's anomaly			1 (0.3)				
Congenital insufficiency of aortic valve		1 (0.3)	1 (0.3)				
Chronic combined systolic (congestive) and diastolic (congestive) heart failure						1 (0.3)	
Tachycardia		1 (0.3)					
***Neurologic diagnoses***	1 (0.3)	1 (0.3)	8 (2)	1 (0.3)	1 (0.3)	1 (0.3)	13
Endocrine	2 (0.5)	2 (0.5)	5 (1.3)		1 (0.3)	2 (0.5)	12
Immunosuppression	1 (0.3)	1 (0.3)	6 (1.5)	1 (0.3)			9
Malignancy	1 (0.3)	1 (0.3)	3 (0.8)				5
Autoimmune diagnoses		1 (0.3)	4 (1)				
Mucocutaneous lymph node syndrome [Kawasaki]	1 (0.3)						
Pre-eclampsia		1 (0.3)					

**Table 6:** Comorbidities analysis of young adults admitted with COVID-19.

	** **	** **		**Fisher's Exact Tests**	
	df	Chi Sq*	p value	Exact Sig. (2-sided)	Exact Sig. (1-sided)
Any comorbidity	1	5.708	0.017	0.018	0.011
Hypertension	1	13.229	0.000	0.001	0.001
Diabetes mellitus	1	10.945	0.001	0.002	0.002
Renal	1	7.733	0.005	0.015	0.015
Cardiac	1	3.970	0.046	0.096	0.056
Hematologic	1	1.660	0.198		
Neurologic	1	1.267	0.260		
Malignancy	1	0.986	0.321		
Endocrine	1	0.940	0.332		
Allergies	1	0.819	0.365		
OSA	1	1.084	0.298		
Pre-Diabetic	1	0.489	0.484		
Other pulmonary disease	1	0.156	0.693		
Asthma	1	0.094	0.759		
Gestational diabetes	1	0.038	0.845		
Immunosuppression	1	0.014	0.906		

*Statistical significance threshold = 3.841 for df = 1 metrics, for p < .05

**Table 7:** Datasets generated for analysis of young adults admitted with COVID-19.

Topic	Metrics
Patient Characteristics	gender, age (whole number), BMI, smoking status or history
Comorbidities	asthma, obstructive sleep apnea (OSA), other pulmonary disease(s), hypertension, diabetes mellitus, pre-diabetic status, pregestational diabetes, renal, cardiac, neurologic, endocrine, immunosuppression, malignancy, hematologic or coagulation problems, immunosuppression
Vitals	temperature, heart rate, SPO2
Laboratories	WBC, neutrophil, lymphocyte, glucose, creatinine, ALT, AST, CRP, D-Dimer, LDH, HbA1c, procalcitonin, ferritin, troponin
Hospital Course and Outcomes	deceased or not as inpatient, length of stay (LOS), admission with cardiac arrest, admission with DKA onset, admission with pregnancy, onset of respiratory distress (including dates/days), newly diagnosed diabetes need for ventilator (incl. dates and days of use), extubation, tracheotomy, post-hospitalization required within a long-term care facility

**Table 8:** Description of binomial process used for analysis of clinical measurements on admission of young adults admitted with COVID-19.

Measure	Binomial details	p value (if < .05)
Temperature	Temperature (< = 98.6 = 0, >98.6 = 1)	0.279
Heart Rate	Heart Rate (70–100)	0.767
SPO2	SPO2 within normal range (95–100)	0.938
WBC	WBC within normal range (4–11)	0.086
Neutrophils (%)	neutrophils% within normal range (13.1–86)	0.560
Lymphocytes (%)	lymphocytes% within normal range (3.0–45.8)	0.112
Sum	All tests within normal range	0.050
HbA1c	tested various levels, from 6.5 to 13, in increments of 0.5 and 1.0	0.050
Glucose	tested for <215 vs ≥215 based upon prior HbA1c test findings	0.000
All values below are tested for within the normal range or not (0 = normal, by gender, 1 = abnormal)		
Creatinine	tested for normal (0) as 0.7–1.30 male, 0.6–1.15 female	0.000
C-reactive protein	tested for varying ranges for binomials: <1, <3, <5, <8 or not	0.029
Procalcitonin	tested for varying ranges for binomials: < .05, < .15, <2 or not	0.000
Ferritin	high end “normal” range defined as: >500 male, >190 female or not	0.115
ALT	tested 10–40 male, 7–35 female, for “normal” (recoded as 0)	0.147
AST	tested 14–20 male, 10–36 female, for “normal” (0)	0.456
D-Dimer	tested for binomial with >250 as abnormal (1)	0.752
LDH	high end “normal” range defined as: 200 male, 190 female	0.630
Troponin		0.330

*Values that are within normal range are coded 0, those outside the normal range are coded 1

**Table 9:** Results of clinical admission analysis using binomial characterization of within or outside of the ranges of the clinical values in Table 7.

						Fisher test	
	n	df	Chi Sq	p	Exact Sig. (2-sided)	Exact Sig. (1-sided)	Significant^#^
Temperature	395	1	0.823	0.364	0.385	0.229	
Heart Rate	395	1	1.648	0.199	0.262	0.129	
SPO2	395	1	0.025	0.874	0.885	0.494	
WBC	387	1	2.596	0.107	0.141	0.076	
Neutrophils (%)	395	1	0.245	0.620	0.653	0.369	
Lymphocytes (%)	395	1	2.531	0.112	0.175	0.078	
Abnormal for >0 tests	395	1	3.744	0.053	0.066	0.034	X
HbA1c*	86	1	4.105	0.043	0.063	0.046	X
Glucose (>215)	395	1	14.655	0.000	0.000	0.000	X
Creatinine	387	1	16.167	0.000	0.000	0.000	X
C-reactive protein	277	1	4.047	0.044	0.050	0.029	X
Procalcitonin (4 groups)	326	3	27.240	0.000	0.000	0.000	X
(2 groups)	326	1	5.987	0.014	0.012	0.012	X
Ferritin	262	1	0.038	0.845	1.000	0.508	
ALT	375	1	1.803	0.179	0.177	0.177	
AST	375	1	1.498	0.221	0.324	0.151	
D-Dimer	282	1	2.110	0.146	0.178	0.098	
LDH	292	1	1.382	0.240	0.607	0.284	
Troponin	206	1	0.377	0.539	0.479	0.479	

*Only the best result is reported, of all tests that were performed: HbA1c >10

#Significance tested for is p < .05

## Results

### Characteristics of the study population

During the timeframe from March 5^th^ 2020 to April 25^th^ 2020 in all New York City public hospitals there was a total of 5,967 patients with a positive COVID-19 test admitted. Those with a positive test in the age range of 18–35 years old and admitted were 769 patients that were captured from the EMR. These charts were then manually reviewed by individually opening each chart and reviewing for a COVID-19 admission. Those who were admitted for reasons other than COVID-19 and incidentally found to be positive, e.g. those admitted for giving birth alone or traumatic injuries, were excluded. This revealed a total of 395 young adults, 18–35 years old, who were admitted for COVID-19 to one of the eleven hospitals of the New York City public health system.

The median age of the young adults, 18–35 years of age, in our study population was 29.9 years (IQR 27–33 years). Hospitalization was more common in males (66.8%) than females (32.9%). Of these 22 were pregnant (5.57%) (Table 1). The patients were racially or ethnically diverse with most common listed as ‘other’ 228 (57.7%) which includes Hispanic or Latino, and black 78 (19.7%), Asian 17 (4.3%). The majority of the patients were non-smokers 253 (64.1%). Smoking status was reviewed in several ways to test for significance. The raw dataset described in Table 1 was evaluated, followed by several regroupings of the data to test for impacts of degree of smoking and simple binomial interpretations of smoking versus non-smoking patients, and never smoked versus history of smoking, past and present. Chi squared analyses were performed for these reviews. None of the groupings demonstrated any significant difference, with Chi squared results ranging from 1.136 to 3.653 (signif = 3.841 for p < .05) and p values ranging from 0.194 to 0.455 (not significant). Fisher’s Exact tests (FE) were performed for two of these four interpretations and provided results of 0.199 (1-sided, 0.397 2-sided for the original dataset, and 0.136 (1-sided), 0.292 (2-sided) for a binomial testing of these data based upon no smoking versus current or past smoking history. Body Mass Index (BMI) demonstrated statistical significance between alive versus deceased groups for nearly all methods tested for these data. The grouping similar to that in Table 1 (BMI evaluated in ranges of 5) produced for results: *X*^2^ = 26.66 (df = 13), p = .014. When BMI is evaluated as a binomial, based upon BMI< = 25 recoded as 0, BMI>25 as 1, it did not demonstrate a significant difference between the two groups (*X*^2^ = 0.557 (df = 1), p = .557, FE = 0.61(1-sided), 0.346(2-sided)). Of the 395 patients, 340 (86.1%) were alive and 55 died (13.1%). Fifty-seven percent of patients had a least one major comorbidity. The most prevalent major comorbidities within this population were pulmonary (77 patients, 19%), DM (68, 17.2%), renal (19, 4.8%), and cardiac (17 patients, 4.3%) (Table 5). In terms of statistical testing and significance of each of these groups (Table 6), findings demonstrate that the most important comorbidities to consider, in descending order by statistical significance, when alive versus deceased patients are compared using a 2x2 Chi Squared (primarily df = 1, critical p = 3.841), the final statistically significant p-values, in ascending order, are: hypertension (n = 44, *X*^2^ = 13.229 (df = 1), p = .00000), diabetes mellitus (n = 68, *X*^2^ = 10,945 (df = 1), p = .001), renal (n, = *X*^2^ = 7,733 (df = 1), p = .005), and cardiac (n, *X*^2^ = 3.97 (df = 1), p = .046). These four statistically significant outcomes were also confirmed using Fisher’s Exact (FE) test. The overall value of performing this test of patient disease or diagnostic history, which includes reporting any or all of the other comorbidities tests as 1 = yes, for one or more comorbidities evaluated as a binomial, resulted in *X*^2^ = 5.708 ((df = 1), p = .017, FE = 0.018 (2-sided), 0.011 (1-sided)) (Table 6). Demonstrating evaluating the basic comorbidities of a patient is an important part of the patient evaluation process when assessing COVID-19 young adult patients.

### Clinical presentation analysis

Young adults clinical presenting mean temperature was 100.2 (IQR 3.0), with a median heart rate was 111 (IQR 22.0) and a mean pulse oximetry (SPO2) 94% (IQR 6.0) (Table 2). Blood pressure which was almost always within normal limits in this age group and was not included. Presenting temperature, heart rate and SPO2 were compared in these patients, between alive versus deceased groups. Each of these was reviewed using the true numeric values for each patient, and an evaluation of distributions of these outcomes relative to theoretical normal ranges for each. For the binomial, patients with numbers > normal range were assigned 1, those within normal range assigned 0. For some non-binomial, true outcome value tests, those values reported as ‘<___’ or ‘>____’ were redefined as a true number to which one whole unit or one half the highest or lowest value was added or subtracted (i.e. >.0001 converted to .00005; >100 to 101). The resulting binomial Chi Squared results demonstrated no significant results for these three metrics, which were: Temp *X*^2^ = 0.823 (df = 1, p = .364), Heart Rate *X*^2^ = 1.648 (df = 1, p = .199), and SPO2 *X*^2^ = 0.025 (df = 1, p = .874) (Tables 8 and 9). A number of the lab panels and inflammatory markers frequently drawn on the initial hospitalization of COVID-19 patients were reviewed (Table 2). The majority had normal WBCs, and the elevated neutrophil (%) and deceased lymphocyte (%) were consistent with viral infection. White blood cell (WBC), neutrophils and lymphocytes were evaluated between alive versus deceased groups, using t-Test and when range of magnitude of scores was high, comparing Log10 values of these amounts. For the binomial, counts were evaluated for whether or not they were in the normal range (0 = yes, 1 = no). The results of this evaluation demonstrated no significant differences for these three outcomes (wbc *X*^2^ = 2.596, df = 1, p = .107; neutr *X*^2^ = 0.245, df = 1, p = .620; lymph *X*^2^ = 2.531, df = 1, p = .112). Evaluating the two groups for absence or presence of any one abnormal labs of the three tested, outcome results were significant to borderline significance, depending upon a researcher’s statistical evaluation logic (*X*^2^ = 3.744, df = 1, p = 0.053, FE = .066(1s), 0.034(2s)). Blood glucose and HbA1c tests were evaluated. HbA1c levels was investigated above and below a certain value, for the range A1c = 6.5 to 13, in increments of 0.5 and 1.0, to determine where statistical significance is approached and then reached. A HbA1c of 9.5 or greater results of Chi Squared (2x2, df = 1) with results centered around *X*^2^_mean_ = 3.864 and p = .05 (range 0.022 to 0.078). An evaluation of glucose results then followed, focusing on glucose levels closely associated with an HbA1c of 9.5 (glucose ~215). This binomial produced a result of *X*^2^ = 14.655 (df = 1), p = .0000) for the alive versus deceased populations. Other similar tests automatically provided in SPSS also supported this highly significant outcome, including FE = 0.000 (1- and 2-sided). Comparing glucose testing results to HbA1c results, glucose (p = .000) was a much stronger indicator of risk related to possible death than HbA1c (p_avg_~0.05 +/- 0.03), with HbA1c of 10.0 or greater serving as the best cut off point for defining the possibility of risk or not (*X*^2^ = 4.105, df = 1, p = .043). Other lab tests demonstrating significant results for alive versus deceased groups were procalcitonin (n = 326, *X*^2^ = 27.240, with df = 3, and *X*^2^ = 5.987, df = 1) and creatinine (n = 387, *X*^2^ = 16.167, df = 1), each resulting in p = 0.000. The evaluation of C-reactive protein (CRP) results (n = 277) demonstrated a significant outcome with *X*^2^ = 4.047, df = 1, p = 0.029. ALT, AST, D-Dimer, ferritin, LDH and troponin did not demonstrate statistically significant differences (Tables 8, 9), although the number who had a high troponin drawn (>0.04, n = 3/206) was small.

### Clinical practice datasets

A more in-depth analysis was done by combining structured and non-structured dataset analysis methods (Table 7). This enabled several new indicators to be developed, focused on unique clinical and/or intervention features of the patient’s care process. Non-structured data are reliable if interrater reliability for these data is consistent and undergoes the same basic reviews from case to case [3, 4]. This process may also be used to confirm, support and be related to the standard structured data forms collected. The data applied to these analyses included patient details (age, gender, pregnant or not), admission characteristics such as respiratory distress, cardiac arrest, diabetes related health (new onset DM, DKA, and history of DM), ventilation and tracheotomy requirements and the period of use for this equipment, inpatient time, notes on whether these resulted in a healthy discharge, a maintained inpatient stay, transfer to a prolonged care setting, or death. An overview of this research logic is provided in (Table 7).

#### General patient characteristics

Gender did not demonstrate any impact of outcomes for alive versus deceased groups (*X*^2^ = 2.53, df = 2 due to one ‘no data’, p = .111). For age range comparisons, two age ranges were defined for this study and assessed for alive versus deceased groups. When evaluated as a five-year increment classification, a similarity between the alive versus deceased groups is inferred by lack of significant p result (*X*^2^ = 1.6218, df = 4, p = .203). A compression of these data into a 10 years age range model produced a significant result when alive versus deceased outcomes were compared (*X*^2^ = 63.794, df = 2, p < .00001). This grouping of patients also allows for a helpful comparison between each group and the rest of the population, for example 20–24 years old versus the rest, 24–29 year old versus the rest, for all possible groupings. Using this latter method to compare and contrast alive versus deceased patients, the high risk of mortality in the twenty-year old patients is revealed; based upon the same testing performed on five-year age groups, to highest risk patients are between 25 and 29.99 years of age in this study population.

#### Clinical features

A series of evaluations focused on special conditions of the patients, including pregnant or not, in a state of cardiac arrest or not, diabetes ketoacidosis or not, and respiratory distress or not. The identification of patients who were pregnant at the time of admission (n = 22), demonstrated no significant results overall, in terms of alive versus deceased (*X*^2^ = 1.710, df = 1, p = .191), but this could be due to the small number of these patients who were evaluated. The cardiac arrest ‘Yes” or 1 entry consisted of a very small number of patients (n = 4), but produced a highly significant outcome (*X*^2^ = 24.980, df = 1, p = 0.000; FE = 0.000(1),0.000(2)). DKA also impacted a relatively small number of patients with a diabetes history (18 DKA patients/44 diabetics, 40.9%), but produced results that lacked statistical significance (*X*^2^ = 0.118, df = 1, p = .731). For comparison, a review of patients with respiratory distress as part of their initial admission characteristics (n = 78) demonstrated very high statistical significance regarding the link of respiratory distress to mortality (*X*^2^ = 236.6842, df = 1, p < .0001). A review of respiratory distress state upon admission (n = 78) relative to patient age also demonstrated significance (*X*^2^ = 13.340, df = 4, p = .010) (S1 Table).

#### Diabetes history and risk

This review employed several methods to analyze the impact of diabetes and diabetes-related medical issues on COVID-19 outcome. The diabetes related risks for this review were identified as: diabetes diagnosis history, the absence or presence of a combination of diagnoses related to diabetes, the diabetes and hypertension and renal disease common associations, admission in DKA, the history of a recent or new diagnosis of this diabetes, the presence or history of a documented gestational diabetes, the diagnosis of a pre-diabetes, as well as the demonstration of abnormal lab results for glucose >215, and/or an abnormal HbA1c results >10 (based upon previous tested of the lab data). The results of this scoring of diabetes risks resulted in values ranging 0 to 4 (maximum score potential = 7). Detailed evaluations of these scores as a 5-point score and a slightly compressed 4-point score (to reduce the small numbers and zero values problems in the equation), resulted in statistically significant outcomes for both of these methods, demonstrating the importance of evaluating the diabetes history (outcomes: 5-point: *X*^2^ = 14.435, df = 4, p = .006, and 4-point: *X*^2^ = 11.045, df = 3, p = .011).

#### Respiratory care requirements

Patients were evaluated for the following respiratory care related actions: ventilation-extubation related period of care, whether they experienced a tracheotomy (group A: n = 3) or not, and the need for reintubation or not (group B: n = 8). These patients were also evaluated for their numbers of days on the ventilator, the activities that ensued during that period of care, and the overall outcome. A total of 78 of the patients in this study were placed on ventilators, averaging 11.41 days (median 7 days; SEM 1.679, IQR = 10). Twenty-five of these patients (32.05%) survived. Three patients underwent tracheotomy (*X*^2^ = 0.489, df = 1, p = .484); eight received additional respiratory care related services including reintubation. Each of these two small groups demonstrated no significant results when compared against the respiratory care patients (n = 78). However, low numbers are perhaps the reason for this lack of significance, for those in either of these groups (n = 3 and 8) were found only in the groups of patients who survived COVID-19 hospitalization; none were in the deceased group. Both the alive and deceased groups each had six patients who were readmitted (*X*^2^ = 1.14, df = 1, p = 0.286). Twenty-nine of the 340 surviving patients (8.5% were placed on BiPAP or CPAP equipment). Patients who were admitted in a documented clinical state of respiratory distress (n = 78) demonstrated a statistically significant outcome likelihood to mortality (X2 = 236.6842, df = 1, p < .0001; Wilcoxon (Gehan) statistic = 95.57, p = 0.000).

### Temporal and survival analysis

#### Temporal information and binomials

The median length of stay for the entire group was 6.0 days (IQR 7 days, average = 7.76) (Table 4). The length of stay in days for alive versus deceased patients were compared, using binomials to defined those that survived or not the following three periods of hospitalization 1–9 versus >9 days (also referred to as less than 10 days or LT10d); less than 21 days (LT21) versus equal to and longer than 21 days; and less than 31 days (LT31days) versus 31 days or more. Analysis used the standard 2x2 Chi squared approach. A highly statistically significant response was found for Time to Death <10 days (*X*^2^ = 18.508, df = 1, p = .000); this risk lessened considerably for the 21 day cut off (*X*^2^ = 3.464, df = 1, p = .063), followed by 31 or more days of hospitalization (*X*^2^ = 2.212, df = 1, p = .137). For this latter process, these binomials are used to define the critical number of days that a patient is on the ventilator, during which, if there is a statistically significant difference in survival rates, potential causes need to be further explored and evaluated using the remaining data already gathered (S1 Table).

#### Survival plots

**Fig 1:**
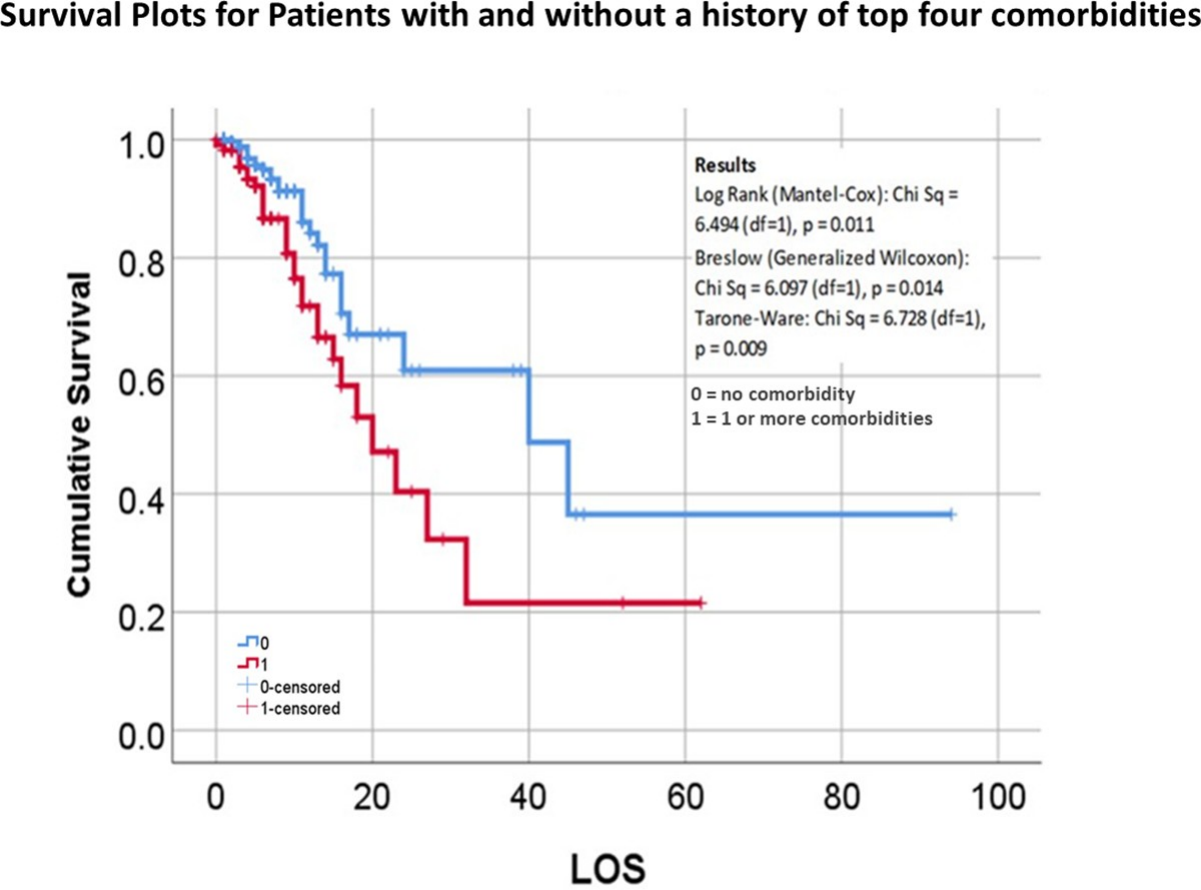
Survival plots for young adults hospitalized with COVID-19 with or without major comorbidity showing statisitcal signifigance (diabetes, hypertension, cardiac, renal), LOS (length of stay). Any of the major comorbidity vs none.

**Fig 2:**
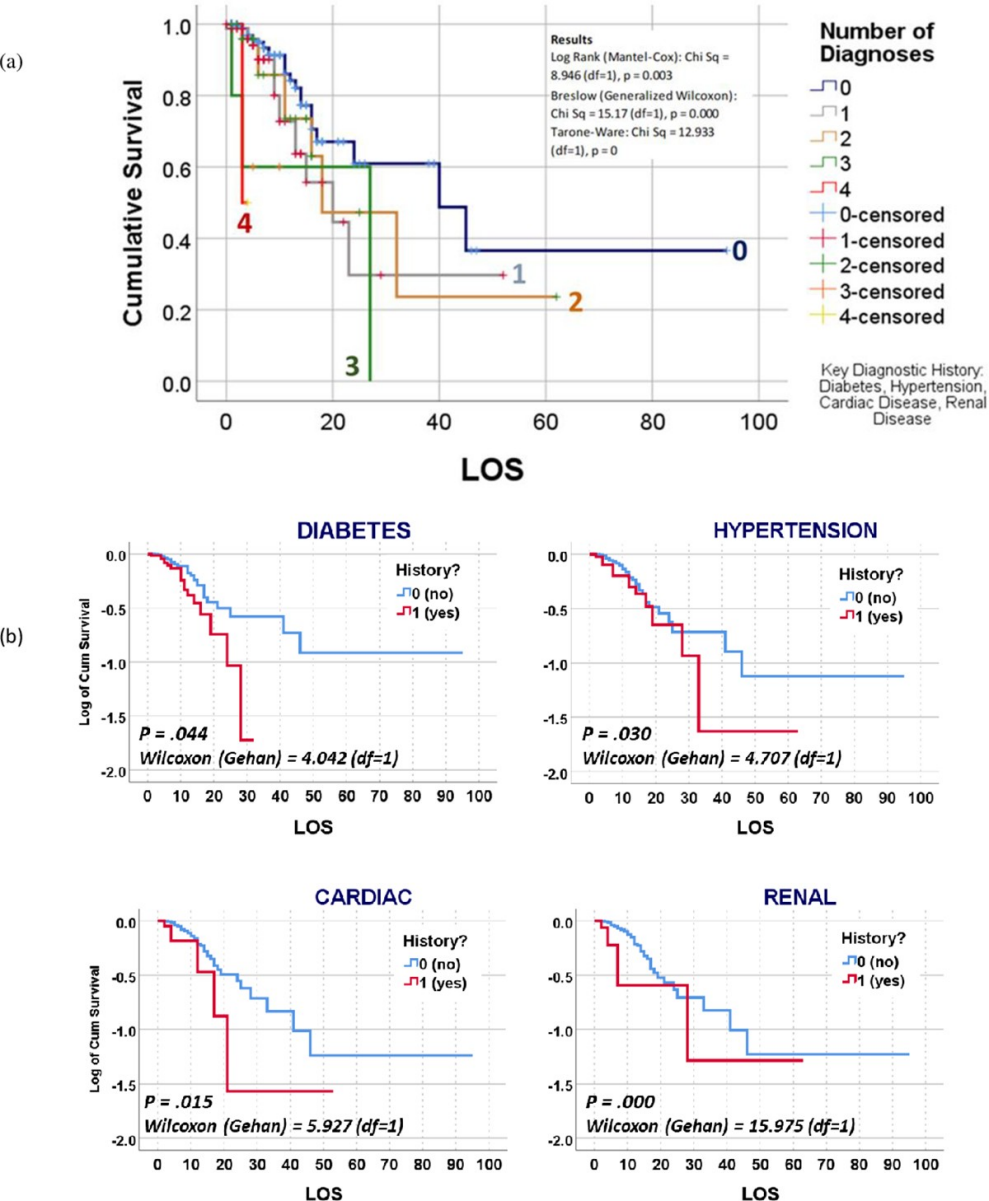
(a) Survival plots for counts of disease history in young adults hospitalized with COVID-19, 0 = none, 1 = diabetes, 2 = hypertension, 3 = cardiac, 4 = renal. LOS (length of stay) (b) Independent plots of these four comorbidities that demonstrated a statistically significant link to COVID mortality (tested for p < .05, df = 1, critical value = 3.841).

**Fig 3:**
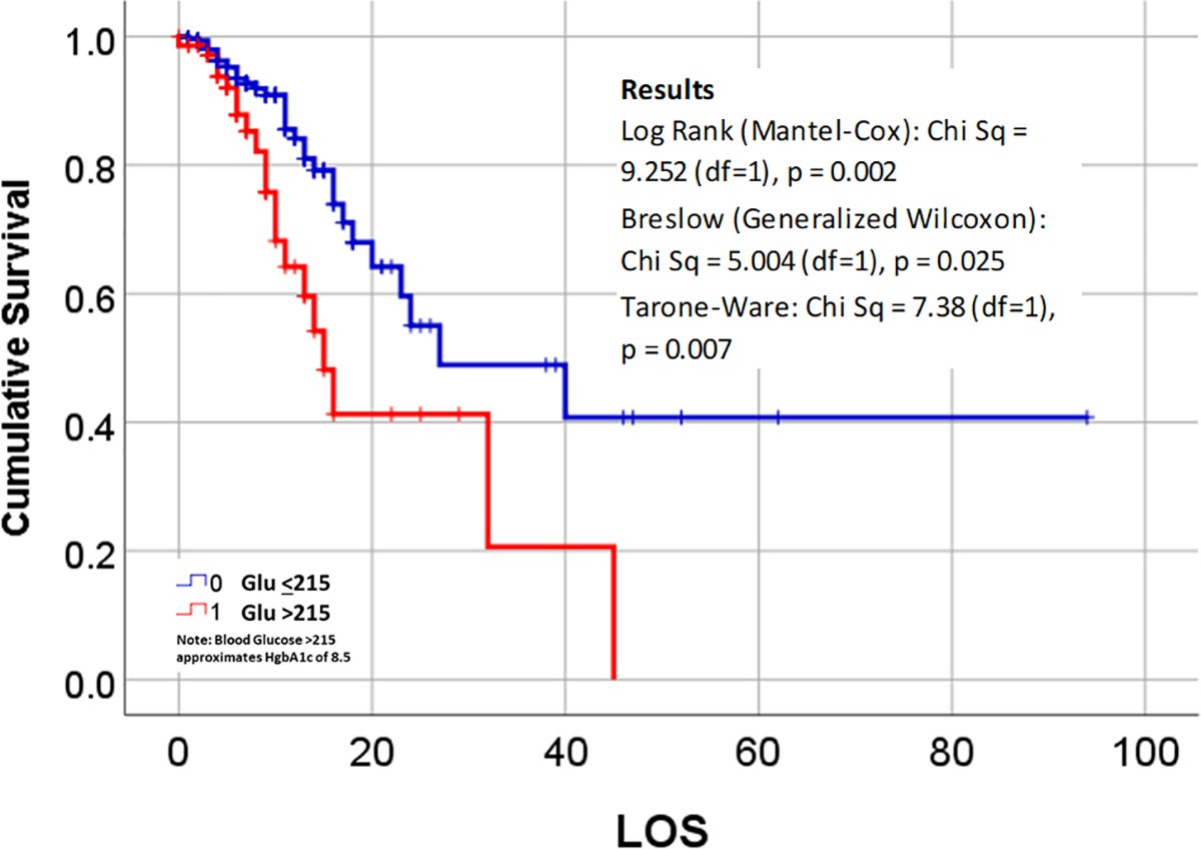
Survival plots for young adults hospitalized with COVID-19 with elevated blood glucose on admission; results of Cox regression analysis of length of stay (LOS) and mortality for patients with Glucose >215 or < = 215.

The last evaluation of the data used Kaplan Meier (KM) survival plots to compare alive and deceased populations. The KM plots were used to re-evaluate all of the previously tested binomials and polynomials demonstrating significant (p < .05) or borderline significant results (0.05 ≤ p < 0.10). The majority of KM reviews focused upon “survival rates” related to patients who: i) required a ventilator or not during their hospital stay, ii) died due to COVID, in relation to patient data, medical history, and hospitalization and treatment data. Survival Rates were compared using the three p-values available in SPSS for evaluating the differences between lines from left to right: Breslow (Generalized Wilcoxon), Tarone-Ware (TW), and Log Rank (Mantel-Cox) testing. The outcomes evaluated this way included age groupings, gender, vitals (normal vs abnormal), lab values (normal vs abnormal), patient history diagnostics groups, smoking history and habit, clinical events (days of inpatient say and ventilator use), actions or findings (diabetic ketoacidosis (DKA), cardiac arrest upon admission (CA)), and several standard risk derived indicators that utilize patient medical history and presentation data, resembling Charlson Score [5, 6], Elixhauser Score [7, 8], and “metabolic syndrome” [9] related risk indicators. Survival plotting demonstrated the following relationships between alive and deceased groups: Kaplan-Meier (KM) plots did not demonstrate significant differences between the followings sets of data gathering and results for vital signs (temperature, SPO2, heart rate), blood testing (WBC, neutrophils and lymphocytes), and labs other than HbA1c and glucose. For risk assessment based upon glucose and HbA1c lab data, testing glucose as ‘GT215 or not’ was successful for survival plots (Wilcoxon (Gehan) = 5.371, p = 0.020) and HbA1c demonstrating detection of significant change starting at HgA1c<8 or not, repeatedly tested up to HbA1c<13 or not; all results successfully defined risk for HgA1c >8–13 (p = 0.014–0.000), not HbA1c>7. Finally, pre-existing disease histories, the most important comorbidities with statistically significant results, in descending order by prevalence, were diabetes (n = 54 deceased/73 diagnosed, 74% tested POS; 98.2% with diabetic history deceased; Wilcoxon p (*Wp*) = .044), hypertension (14/44, 32% POS, 25.5%; *Wp* = 0.030) and then renal (6/16, 37.5% POS, 11%; *Wp* = 0.000) and cardiac (6/21, 28.6% POS, 11%; *Wp* = 0.015) disease. KM plots with statistically significant result for diabetes and for the four defined statistically significant indicators are shown in Figs 1–3.

## Discussion

Throughout the early stages of the COVID-19 pandemic, studies have focused on the older populations and risks [10–13]. There is currently limited information regarding youth and young adults, or in public health systems, during the pandemic [14, 15]. This is likely due to lack of symptomatology on behalf of children [16, 17] and lack of reporting or presentation at the time by young adults [18]. Severe consequences of a COVID-19 infection include respiratory distress [19], need for ventilator [20], tracheotomy [21], and sequential organ failure [22, 23]. In older adults chronic diseases linked to COVID-19 fatality has been diabetes [24, 25] hypertension [26], cardiovascular disease [27, 28], renal [29] or renal-endocrine [30–32] disease.

It has been shown that age and co-morbidities influence outcomes of those admitted with COVID-19. Previous studies demonstrated that older patients were more likely to have a severe COVID-19 [33, 34]. In this analysis of young adults hospitalized with COVID-19, it is important to consider features which may impact younger populations that overlap with older populations. We demonstrate that young adults in addition to older patients represent a portion of those with severe outcomes. Young adults admitted with COVID-19 in New York City public hospitals had an overall mortality 13.1%. Isolated studies combining all ages in areas around the City report mortality from 10–21% [2, 35]. As age and comorbidities may impact disease severity of COVID-19, potential regional variations in health may underlie the impact on specific populations. It is likely that patients treated in hospitals in New York City had a higher average body mass index, greater prevalence of hypertension, diabetes, and chronic pulmonary disease than those characterized in Italy and China [36]. When our overall disease and diagnosis history was reviewed in this study 57.9% had at least one major comorbidity. Young adults who died without a major comorbidity were relatively rare, representing only 3.8% of those admitted. The most prevalent major comorbidities within our population in this study were found to be pulmonary (19%), DM (17.2%), renal (4.8%), and cardiac (4.3%) and were shown to have increased risk in respect to mortality. A further notable finding in our analysis of young adults admitted with COVID-19 also suggests that elevated blood glucose on admission and elevated HbA1c tests are associated with increased mortality risk. To date, there is currently limited information of the temporal course and survival for those hospitalized with COVID-19. These outcomes may also be used support other standard methods in use to define mortality risk based upon common data developed for COVID-19 patients, such as logistic regression, t-testing and chi squared analyses [37]. Such analysis is useful to identify important indicators in defining high risk COVID-19 patients, such as with age and medical history. Our temporal and survival analysis define four comorbidity risks that currently demonstrate a statistically significant link to COVID-19-related mortality in young adults. We believe temporal investigations are important for further study of longer-term effects of COVID-19 in those with disease severe enough for hospitalization. Kaplan Meier survival analysis can be used to demonstrate important findings demonstrated by temporal data. Further investigation may help characterize those who recover quickly versus those who have severe disease requiring prolonged hospitalization.

Recent findings suggest there may current be a shift in transmission rates in United States cases to younger individuals [38]. As the pandemic continues, there is a need for additional investigations into COVID-19 morbidity and mortality reports in the U.S. and findings specific to young adults [39]. This report, which we believe to be the largest specific analysis of hospitalized young adults in the U.S. to date, provides insight into this age group hospitalized with COVID-19. There were several limitations in our study that might create bias. First, it was retrospective and a single health care system study of patients admitted to the hospitals in New York City. Additional data from multiple health care systems may be better to assess the total scope of COVID-19 in this age group. As current studies demonstrate different country and regional mortality, further analysis may help better define disease patterns. This study demonstrates that existing co-morbidities and disparities in the populations such as in New York City as well as those in public health are important factors to consider in the pandemic. Based upon the nature of the health care system in which this study took place, the patients in this study are comprised mostly of low socioeconomic status groups with particular race-ethnicity backgrounds, and represent a considerable portion of the high-risk population typically reported for this region. This report describes the burden of young adults with COVID-19 infection in New York City health system and confirms that severe illness in young adults is significant but far less frequent than older aged individuals. Prehospital comorbidities appear to be an important factor in young adults. Additionally, these findings suggest the need for larger and more extensive studies of young adults with COVID-19 infection as well as those with severe disease in the absence of known comorbidities.

## Supporting information

S1 Table Summary results of datasets and survival analysis. (DOCX)
