# Supplementary material for: Characteristics, comorbidities and survival analysis of young adults hospitalized with COVID-19 in New York City
Source: PLoS One. 2020 Dec 14;15(12):e0243343. doi: 10.1371/journal.pone.0243343 (PMC7735602; doi:10.1371/journal.pone.0243343)
Supplement: S1 Table — (DOCX) [file pone.0243343.s001.docx]

**S1 Table. Summary results of datasets and survival analysis**

|  |  |  |  |  | **Fisher test** |  |
| --- | --- | --- | --- | --- | --- | --- |
| **Metric** | **N notes** | **df** | **Chi Sq** | **p** | **Exact Sig. (2-sided)** | **Exact Sig. (1-sided)** |
| Age range (5 yrs) | var; 5 yr groups; detailed model | 4 | 1.6218 | 0.203 |  |  |
| Age group (10 yrs) | var; 10 yr groups; best model | 2 | 63.794 | <.00001 |  |  |
| Gender | 130 female, 264 male, 1 unknown | 2 | 2.532 | 0.112 |  |  |
| Pregnancy (1=yes) | 22 were pregnant; not further reviewed due to low n | 1 | 1.710 | 0.191 | 0.338 | 0.160 |
| Respiratory distress on admission* and Age | The only analysis not evaluating Alive vs Deceased; 78 had Resp Distress; this compares them using Age Range data | 4 | 13.340 | 0.010 |  |  |
| Respiratory distress upon admission* | 78-yes, 317-no | 1 | 236.684 | <.0001 |  |  |
| Cardiac arrest upon admission* | 4-yes, 391-no# | 1 | 24.980 | 0.000 | 0.000 | 0.000 |
| New onset diabetes noted^ | New DM, 3-yes, 392-no# | 1 | 18.687 | 0.000 | 0.003 | 0.003 |
| DKA upon admission (yes=1) | DKA, 18-yes, 377-no | 1 | 0.118 | 0.731 | 0.726 | 0.470 |
| Overall count of Diabetes Risks | Any DM risk: 44-yes, 120-no, 231-no diagnosis indicators | 4 | 14.435 | 0.006 |  |  |
| Diabetes Risks Scaled down | Any DM risk: 44-yes; merging the 2 highest risk groups | 3 | 11.045 | 0.011 |  |  |
| Three Risk groups: DiabRisk012 | Any DM risk: only testing 44-yes, 120-no | 2 | 0.147 | 0.929 |  |  |
| Diabetes Risk Binomial (yes=1) | Any DM risk: only testing 44-yes, 120-no | 1 | 0.130 | 0.718 | 1.000 | 0.529 |
| Extubate minus ventilator date | Numbers of Days, independently; 78 eval | 26 | 27.994 | 0.359 |  |  |
| Tracheotomy outcome | 3 tested; probably too low# | 1 | 0.489 | 0.484 | 1.000 | 0.637 |
| Ventilator reintubation needed | 8 tested; probably too low# | 1 | 1.321 | 0.250 | 0.606 | 0.298 |
| LT10d** | Survival data | 1 | 18.508 | 0.000 | 0.000 | 0.000 |
| LT21d** | Survival data | 1 | 3.464 | 0.063 | 0.103 | 0.069 |
| LT31d** | Survival data | 1 | 2.212 | 0.137 | 0.150 | 0.150 |
|  |  |  |  |  |  |  |
| *clinical patient presentation |  |  |  |  |  |  |
| ^noted in clinical course of patient |  |  |  |  |  |  |
| #unreliable due to low n |  |  |  |  |  |  |

** The length of stay in days for Alive versus Deceased patients were compared using binomials to defined those that survived or not the following three periods of hospitalization 1-9 versus >9 days (also referred to as less than 10 days or LT10d); less than 21 days (LT21) versus equal to and longer than 21 days; and less than 31 days (LT31days) versus 31 days or more.
